# BAF-L Modulates Histone-to-Protamine Transition during Spermiogenesis

**DOI:** 10.3390/ijms23041985

**Published:** 2022-02-11

**Authors:** Chao Huang, Huan Gong, Bin Mu, Xinting Lan, Chengcheng Yang, Jinlong Tan, Wentao Liu, Yuanfeng Zou, Lixia Li, Bin Feng, Xia He, Qihui Luo, Zhengli Chen

**Affiliations:** 1 Laboratory of Experimental Animal Disease Model, College of Veterinary Medicine, Sichuan Agricultural University, Chengdu 611130, China; gh3107@163.com (H.G.); mb616@163.com (B.M.); lxt9091@163.com (X.L.); ycc0428@outlook.com (C.Y.); tjlong14@163.com (J.T.); liuwt1986@126.com (W.L.); lqhbiology@163.com (Q.L.); 2 Key Laboratory of Animal Disease and Human Health of Sichuan Province, College of Veterinary Medicine, Sichuan Agricultural University, Chengdu 611130, China; yuanfengzou@sicau.edu.cn (Y.Z.); lilixia905@163.com (L.L.); 3 Animal Nutrition Institute, Sichuan Agricultural University, Chengdu 611130, China; fengbin@sicau.edu.cn (B.F.); hx701228@126.com (X.H.)

**Keywords:** BAF-L, spermiogenesis, histones, protamines, male infertility

## Abstract

Maturing male germ cells undergo a unique developmental process in spermiogenesis that replaces nucleosomal histones with protamines, the process of which is critical for testicular development and male fertility. The progress of this exchange is regulated by complex mechanisms that are not well understood. Now, with mouse genetic models, we show that barrier-to-autointegration factor-like protein (BAF-L) plays an important role in spermiogenesis and spermatozoal function. BAF-L is a male germ cell marker, whose expression is highly associated with the maturation of male germ cells. The genetic deletion of BAF-L in mice impairs the progress of spermiogenesis and thus male fertility. This effect on male fertility is a consequence of the disturbed homeostasis of histones and protamines in maturing male germ cells, in which the interactions between BAF-L and histones/protamines are implicated. Finally, we show that reduced testicular expression of BAF-L represents a risk factor of human male infertility.

## 1. Introduction

Spermatozoa are generated through spermatogenesis, the controlled progress of which is essential for testicular homeostasis and male fertility [1,2]. Spermatogenesis contains a sequence of highly orchestrated developmental steps of spermatogenic lineage, involving mitotic expansions, meiotic divisions, and the process of spermiogenesis [3]. Male germ cells are unique in that they will undergo morphological transformations including nuclear condensation, acrosome formation, cytoplasm removal, and tail elongation during spermiogenesis [4]. Among these processes, the replacement of nucleosomal histones with protamines in elongating spermatids is critical for the protection of the genome from DNA damages and the effective genetic transmission [5]. Disruption in the histone/protamine exchange results in genome package and nuclear condensation defects and will also causes male subfertility [6,7]. However, the mechanisms regulating histone/protamine exchange in spermiogenesis are complex and still not well understood.

The barrier-to-autointegration factor (BAF), a highly conserved and predominantly nuclear-located protein, is reported to function in chromatin organization such as chromosome segregation and nuclear assembly [8]. BAF is also involved in many other cellular processes, including viral infection, genes transcription, DNA damage, nuclear rupture repairment and gonad development [9,10,11,12]. In mammalian organisms, BAF has a homologous gene termed BAF-Like (BAF-L), which shows a 53% similarity in the protein sequence with BAF in humans [13]. While BAF is widely expressed, nuclear-located BAF-L seems to be most abundant in testes and is present in human spermatozoa [13,14]. In addition, the corelated expression of BAF-L with testicular development, as well as the modified expression pattern of BAF-L in the spermatozoa of globozoospermic patients, suggests important roles of BAF-L in spermatogenesis and male fertility [15,16]. Therefore, an animal model with genetically modified BAF-L could bring about values for evaluating functions of testicular BAF-L. In this study, we assessed the correlation of mouse BAF-L expression with spermatogenesis and used loss-of-function mouse models to examine the role of BAF-L in testicular homeostasis and sperm function. Finally, we linked reduced spermatozoal BAF-L to human male infertility.

## 2. Results

### 2.1. Reduced Expression of BAF-L Is Related to Human Asthenozoospermia

**Figure 1:**
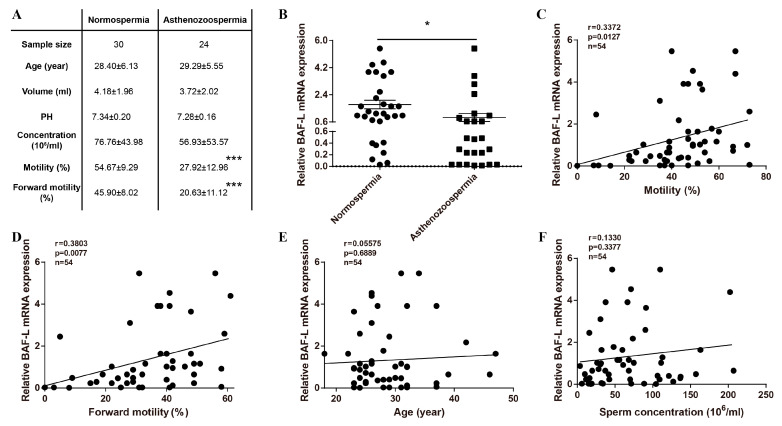
Reduced BAF-L mRNA expression corelates with human asthenozoospermia. (**A**) Seminal characteristics of the study subjects. *** *p* < 0.001 by two-tailed Student’s *t*-test. (**B**) qRT-PCR shows decreased spermatozoal BAF-L expression in asthenozoospermic patients. Error bars indicate SEM. * *p* < 0.05 by two-tailed Student’s *t*-test. (**C**,**D**) Linear regression analysis showed positive correlation of spermatozoal BAF-L expression with sperm motility/forward motility. (**E**,**F**) Linear regression analysis showed no correlation between spermatozoal BAF-L expression with patient age/sperm concentration.

The testis is a unique organ where a significant number of genes are specific or prominently expressed, although the roles of most are not well defined, and BAF-L is such a gene. We focused on BAF-L during the course of screening risk factors for male infertility with spermatozoa from patients attending sterility evaluation (Figure 1A). We observed a decreased expression of BAF-L in the spermatozoa of asthenozoospermic subjects compared with normospermic ones (Figure 1B). To evaluate the correlation of BAF-L expression with different sperm parameters, a linear regression analysis was performed. Results show that the expression of spermatozoal BAF-L correlated with the sperm motility/forward motility (Figure 1C,D). No correlation was observed between the expression of spermatozoal BAF-L and the sperm concentration and the subject’s age (24 to 46 years old) (Figure 1E,F). These data reveal spermatozoal BAF-L is implicated in some forms of male infertility and suggest a role of BAF-L in regulating spermatozoal function.

### 2.2. BAF-L Is a Male Germ Cell Marker That Corelates with Spermatozoal Maturation

**Figure 2:**
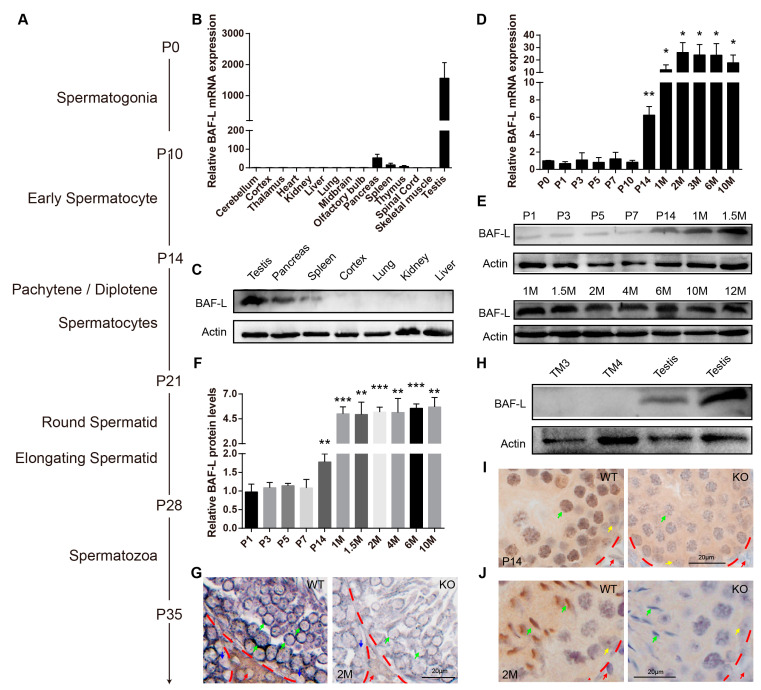
BAF-L is a germ cell marker that corelates with male germ cells’ maturation. (**A**) Time course of mouse spermatogenesis. (**B**,**C**) Tissue expression profile of mouseBAF-L analyzed by qRT-PCR and Western blots in 8-week-old WT mice. Error bars indicate SEM. *n* = 3. (**D**) qRT-PCR shows the expression profile of BAF-L mRNA during testicular development. Error bars indicate SEM. * *p* < 0.05, ** *p* < 0.01 by two-tailed Student’s *t*-test, indicating the statistic differences between P0 and the other timepoint. *n* = 4. (**E**,**F**) Western blots and quantification show the expression profile of BAF-L protein during testicular development. Error bars indicate SD. ** *p* < 0.01, *** *p* < 0.001 by two-tailed Student’s *t*-test, indicating the statistic differences between P0 and the other timepoint. *n* = 3. (**G**) In situ hybridization displays germ cells’ expression of BAF-L in testis. Green arrows indicate the germ cells, blue and red arrows indicate Sertoli and Leydig cells, respectively. (**H**) Western blots show no expression of BAF-L protein in TM3 and TM4 cells. (**I**,**J**) Immunohistochemical images display the localization of BAF-L (brown color) in the nucleus (blue color stained by hematoxylin) of male germ cells, especially in elongating/elongated male germ cells. Green arrows indicate germ cells, yellow arrows indicate Sertoli cells, and red arrows indicate Leydig cells.

In mice, spermatozoa are generated by regular and continuous spermatogenic waves in an age-dependent manner (Figure 2A) [17,18]. To understand the roles of BAF-L in spermatogenesis, we first examined the temporal and spatial expression profile of BAF-L in mouse testes. We found BAF-L mRNA expressed in the thymus, spleen, pancreas, and most abundantly in testis (Figure 2B), and this was further validated by Western blots (Figure 2C). The testicular BAF-L mRNA and protein levels keep increasing in parallel with the first spermatogenic wave beginning from two weeks after birth and remain high throughout adulthood (Figure 2D–F). In situ hybridization showed that BAF-L mRNA was strongly expressed within the seminiferous tubules, especially in the germ cells of adult testes, and fewer signals were observed in Leydig or Sertoli cells (Figure 2G), revealing BAF-L as a male germ cell marker. Consistent with this, no immunoblotting evidence of BAF-L was observed in mouse Leydig or Sertoli cell lines (TM3 or TM4 cells), respectively (Figure 2H). These findings were further validated by immunohistochemistry staining with a BAF-L-specific antibody, showing BAF-L was present in male germ cells both at the juvenile and adult age (Figure 2I,J). Most importantly, we noticed that at the adult age, BAF-L was highly concentrated in the nucleus of elongating/elongated spermatids (Figure 2J). This is also consistent with the finding that increased expression of BAF-L in mouse testes reaches its peak around 4 weeks of age (Figure 2D–F), when elongating/elongated spermatids make up most of the testicular cells [17]. This germ cell’s expression and spermatogenic-wave-associated characteristics of BAF-L suggest its role in spermatozoal maturation and function.

### 2.3. BAF-L Does Not Affect the Early Progress of Spermatogenesis

**Figure 3:**
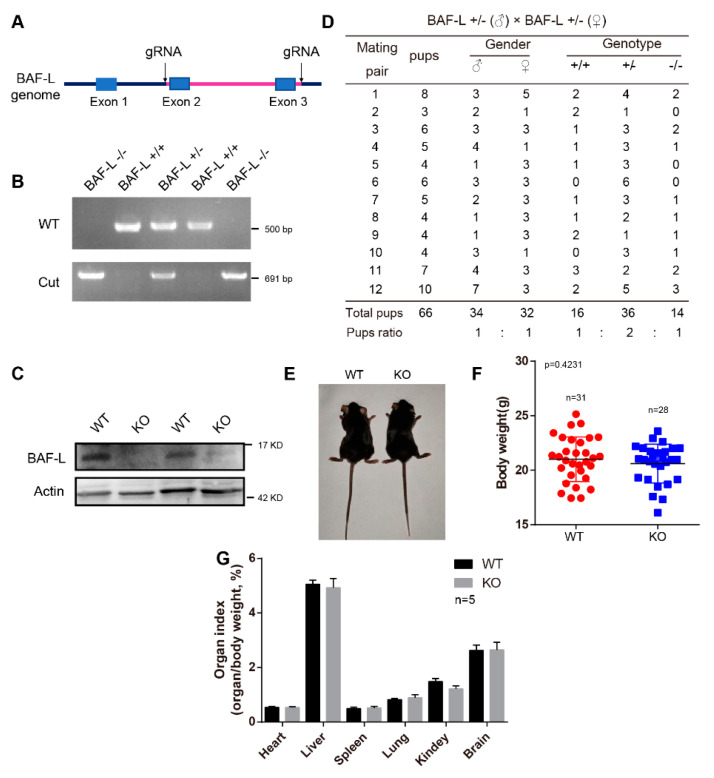
Generation of BAF-L knockout mice. (**A**) Overview of the targeting strategy for generating BAF-L knockout mice Red line indicates the deleted region. (**B**) Genotyping PCR for the validation of BAF-L knockout mice. (**C**) The absence of BAF-L protein is validated by Western blots. (**D**) Quantification shows the number and frequency of offspring of each genotype produced by crossing BAF-L+/− mice. Genotypes of the pups born were predicated with expected Mendelian frequencies. (**E**) Photograph of adult BAF-L WT and KO mice. (**F**) Quantification shows normal bodyweight of 8-week-old BAF-L KO mice. Error bars indicate SD. *n* = 31 for WT and *n* = 28 for KO. (**G**) Quantification reveals normal organ index of 8-week-old BAF-L KO mice. Error bars indicate SD. *n* = 5.

To further examine the biological functions of BAF-L, we generated a BAF-L knockout mouse (BAF-L −/−, KO), in which the exon 2 and 3 of BAF-L were deleted (Figure 3A). The genotypes of the offspring were evaluated by PCR and the absence of the BAF-L protein was validated by immunoblotting (Figure 3B,C). We found that BAF-L KO mice could be born with an expected Mendelian ratio and displayed normal postnatal viability (Figure 3D). The bodyweights of adult KO mice were comparable with WT controls (Figure 3E,F), and no differences in the organ index of some important organs were observed in BAF-L KO mice (Figure 3G). These results suggest that BAF-L is not required for embryonic viability, which is different from BAF, the downregulation of which causes embryonic lethality [19].

**Figure 4:**
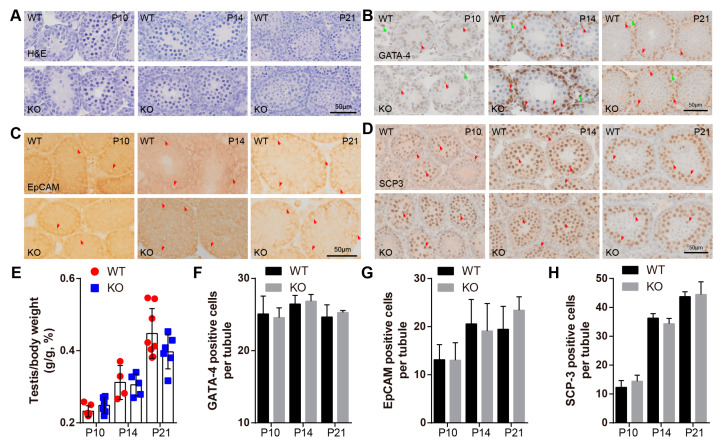
BAF-L does not affect the early progress of mouse spermatogenesis. (**A**) Representative images of H&E staining show normal histological structure of BAF-L KO testis at the age of P10, P14, and P21. (**B**,**F**) Representative images of GATA-4 labeling and quantification show comparable Sertoli and Leydig cells in BAF-L KO testis at the age of P10 (*n* = 4), P14 (*n* = 3), and P21 (*n* = 3). Error bars indicate SD. Red arrows indicate Sertoli cells, and green arrows indicate Leydig cells. (**C**,**G**) Representative images of EpCAM labeling and quantification show comparable spermatogonia cells in BAF-L KO testis at the age of P10 (WT, *n* = 4; KO, *n* = 5), P14 (*n* = 4), and P21 (WT, *n* = 4; KO, *n* = 5). Error bars indicate SD. Arrows indicate spermatogonia cells. (**D**,**H**) Representative images of SCP3 labeling and quantification show comparable spermatocytes in BAF-L KO testis at the age of P10 (WT, *n* = 3; KO, *n* = 4), P14 (*n* = 3) and P21 (*n* = 3). Error bars indicate SD. Arrows indicate spermatocytes. (**E**) Quantification shows no difference in the ratio of testis and bodyweight of BAF-L KO mice at the age of P10 (WT, *n* = 7; KO, *n* = 6), P14 (WT, *n* = 4; KO, *n* = 5) and P21 (WT, *n* = 8; KO, *n* = 6). Error bars indicate SD.

Spermatozoa are generated by repeated spermatogenic waves, which consist of three highly coordinated and complex steps [20]. First, spermatogonial stem cells undergo mitotic divisions and differentiation to generate spermatocytes; second, spermatocytes meiosis results in the formation of haploid spermatids that enter the third step, termed spermiogenesis, in which a series of morphological transformations progress [21]. During three weeks postnatal, when the first two steps of the initial spermatogenic wave were progressing, we found that BAF-L KO testes appeared to be morphological normally, as indicated by hematoxylin–eosin staining (H&E) (Figure 4A). The testis index (the ratio of testis and bodyweight) was also comparable between BAF-L WT and KO mice at the age of P10, P14, and P21 (Figure 4E). We further analyzed the homeostasis of different cell types during this period of testicular development, observing no changes in the cell numbers of Leydig or Sertoli cells (GATA-4-labeled) (Figure 4B,F), spermatogonia stem cells (EPCAM-labeled) (Figure 4C,G), or spermatocytes (SCP-3-labeled) (Figure 4D,H) in the testes of P10-, P14-, and P21-day-old BAF-L KO mice. These data reveal that BAF-L is not required for the early progress of spermatogenesis. 

### 2.4. BAF-L Deficiency Affects the Progress of Spermiogenesis

**Figure 5:**
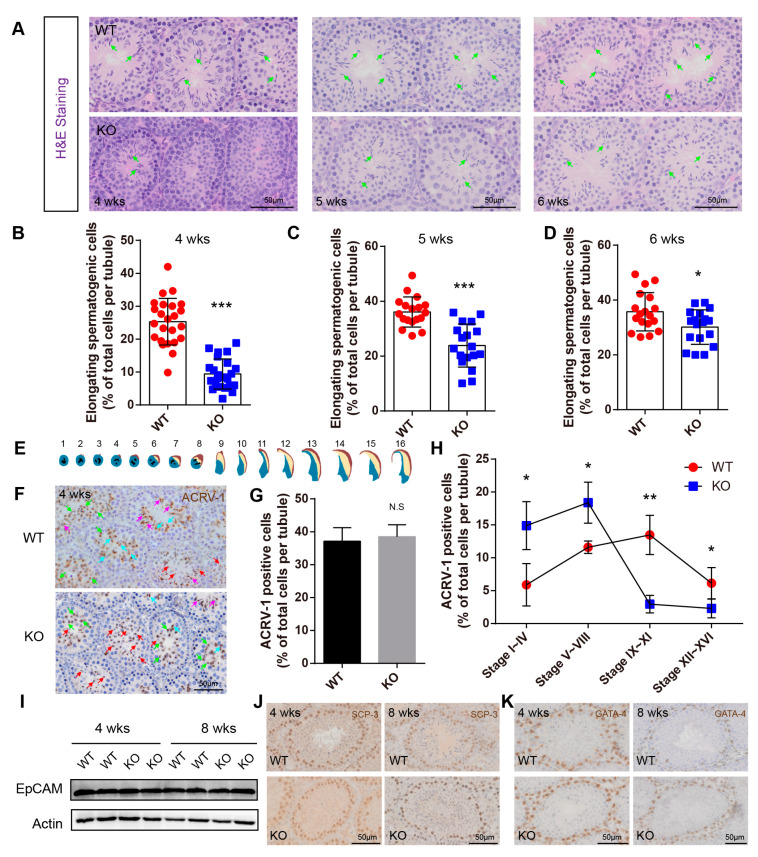
BAF-L deficiency causes defects in spermiogenesis. (**A**–**D**) Representative images of H&E staining and quantifications show decreased numbers of elongating germ cells in the BAF-L KO testis at the age of 4 weeks, 5 weeks, and 6 weeks. Error bars indicate SD. * *p* < 0.05, *** *p* < 0.001 by two-tailed Student’s *t*-test. Green arrows indicate elongating/elongated spermatids. (**E**) Illustration for the 16 developmental stages of mouse spermatids. (**F**) Representative images of ACRV-1 labeling in 4-week-old BAF-L KO testis. Red arrows indicate stage 1–4, green arrows indicate stage 5–8, blue arrows indicate stage 9–11, and purple arrows indicate 12–16. (**G**) Quantification shows comparable ACRV-1-positive cells in 4-week-old BAF-L KO testis. Error bars indicate SD. N.S by two-tailed Student’s *t*-test. *n* = 3 for WT and *n* = 4 for KO. (**H**) Quantification shows a delay in spermiogenic progress in 4-week-old BAF-L KO testis. Error bars indicate SD. * *p* < 0.05, ** *p* < 0.01 by two-tailed Student’s *t*-test. *n* = 3 for WT and *n* = 4 for KO. (**I**) Western blots show no difference in EpCAM protein level in 4-week-old and 8-week-old BAF-L KO testis. (**J**,**K**) Representative images of SCP3 and GATA-4 labeling in 4-week-old and 8-week-old BAF-L WT/KO testis.

While no defects were found in the early progress of spermatogenesis, we observed abnormal spermiogenesis in BAF-L KO mice. Around four weeks of age, haploid spermatids in WT mice begin to transform into elongating spermatids (Figure 5A). However, we found that in BAF-L KO mice, the number of elongating spermatids was less than that of WT controls at this time and till the end of the first spermatogenic wave (six weeks of age) (Figure 5A–D). To further evaluate this, immunochemistry staining of ACRV-1 was performed to label the acrosome of spermiogenic germ cells, which could be divided into sixteen steps (step 1–16) defined by acrosome structure and nuclear morphology changes in developing spermatids [22] (Figure 5E). We found that the ratio of testicular ACRV-1-positive cells and total spermatogenic cells was comparable between BAF-L KO mice and WT controls at four weeks of age (Figure 5F,G), indicating normal developmental progress from spermatogonia to haploid spermatids. However, a significantly increased ratio of step 1–8 round spermatids and decreased ratio of step 9–16 elongating spermatids were present in four-week-old BAF-L KO testes (Figure 5H), suggesting a role of BAF-L in promoting the progress of spermiogenesis, especially in the elongating process. This effect of BAF-L seems to be intrinsic to spermiogenic germ cells, as we observed no changes in the numbers of Leydig or Sertoli cells, spermatogonia, or spermatocytes, as indicated by immunolabeling or immunoblotting with their specific cell markers (Figure 5I–K). Besides, this is also consistent with the testicular expression profile of BAF-L, which sharply increases around four weeks and reaches its peak before six weeks after birth, the period at which spermiogenesis occurs. These results display the critical importance of BAF-L in regulating spermiogenesis.

### 2.5. BAF-L Regulates Histone–Protamine Exchange in Spermiogenesis 

**Figure 6:**
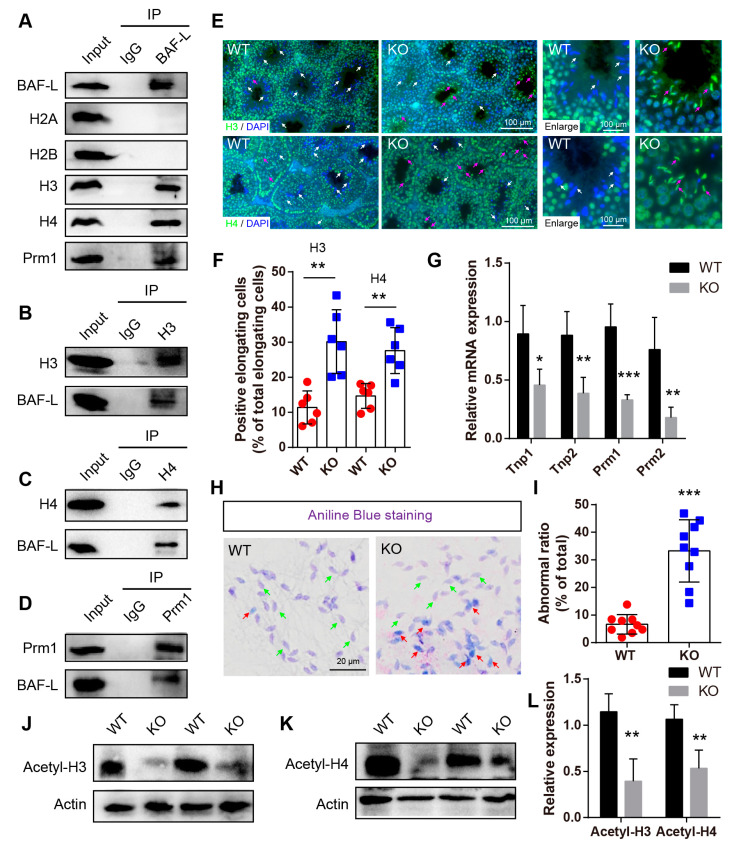
BAF-L is implicated in the exchange of histones and protamines in spermiogenesis. (**A**) Immunoprecipitation analysis performed with BAF-L antibody displays the interaction of BAF-L with histones H3 and H4 and protamine 1 but not histones H2A nor H2B. (**B**) Immunoprecipitation analysis performed with histone H3 antibody displays the interaction of BAF-L with histone H3. (**C**) Immunoprecipitation analysis performed with histone H4 antibody displays the interaction of BAF-L with histone H4. (**D**) Immunoprecipitation analysis performed with protamine 1 antibody displays the interaction of BAF-L with histone protamine 1. (**E**,**F**) Immunofluorescence staining and quantification show increased labeling of histones H3 and H4 in elongating/elongate spermatids of 5-week-old BAF-L KO testis. White arrows indicate normal elongating/elongated spermatids, while purple arrows indicate elongating/elongated spermatids with histones. Error bars indicate SD. ** *p* < 0.01 by two-tailed Student’s *t*-test. *n* = 6 for WT and *n* = 5 for KO. (**G**) qRT-PCR shows decreased mRNA expression of Tnp1, Tnp2, Prm1, and Prm2 in 5-week-old BAF-L KO testis. Error bars indicate SEM. * *p* < 0.05, ** *p* < 0.01, *** *p* < 0.001 by two-tailed Student’s *t*-test. *n* = 4. (**H**,**I**) Representative images of aniline blue staining and quantification show increased ratio of spermatozoa with blue stain in BAL-L KO spermatozoa. Green arrows indicate normal spermatozoa, while red arrows indicate spermatozoa with histones. Error bars indicate SD. *** *p* < 0.001 by two-tailed Student’s *t*-test. (**J**–**L**) Western blots and quantification show decreased protein level of Acetyl-histone H3 and Acetyl-histone H4 in BAF-L KO testis. Error bars indicate SD. ** *p* < 0.01 by two-tailed Student’s *t*-test. *n* = 4.

As we have shown that BAF-L concentrates in the nuclei of male germ cells (Figure 2I,J), and it does not bind to DNA [13], we then asked if BAF-L physically interacts with nucleoproteins in male germ cells. To test this, an immunoprecipitation assay was first performed with a BAF-L antibody using testis lysates, followed by immunoblots with specific antibodies of histones H2A, H2B, H3, and H4. We found that BAF-L interacted with histones H3 and H4, but interestingly not with histones H2A and H2B in vivo (Figure 6A). These results were validated by a further immunoprecipitation assay that was immunoprecipitated with anti-H3/H4 antibodies and then immunoblotted with an anti-BAF-L antibody (Figure 6B,C). Given that histones in male germ cells are replaced by protamines during spermiogenesis and a high expression of BAF-L is associated with spermiogenesis, we then evaluated whether BAF-L was also a protamine-interacting protein, finding a direct interaction between BAF-L and protamine 1 in vivo (Figure 6A,D). These data reveal that the nuclear location of BAF-L results from its interaction with nucleoproteins and suggests a role of BAF-L in regulating the homeostasis or function of nucleoproteins.

The histone–protamine exchange is one of the most important processes during spermiogenesis. As BAF-L interacts with both histones and protamine, we wondered if these interactions were implicated in the replacement of protamines with histones. To address this, immunofluorescence staining with antibodies of histones H3 and H4 was performed. We found that, at sexual maturity age, histones H3 and H4 were highly expressed from spermatogonia to round spermatids but were almost absent in elongating/elongated spermatids in WT mice (Figure 6E). However, in KO mice, widespread immunofluorescence labeling of histone H3 and H4 was observed in elongating/elongated spermatids (Figure 6E,F), suggesting defects in the exchange process of histones and protamines. Consistent with this, the qRT-PCR displayed robust decreased expressions of protamines (Prm1 and Prm2), as well as the transition proteins (Tnp1 and Tnp 2) in BAF-L KO mice (Figure 6G) [23]. Moreover, aniline blue staining, which evaluates spermatozoal chromatin defects related to their nucleoprotein content, revealed much more blue stain in BAF-L KO spermatozoa (Figure 6H,I), indicating the presence of lysine-rich histones in BAF-L KO spermatozoal nuclei [24]. The defects in the histones–protamines exchange may corelate with reduced acetylation of histones H3/H4 (Figure 6J–L), which is an important post-translational modification of histones that is critical for regulating spermiogenesis [25]. All these results display a critical role of BAF-L in spermiogenesis in regulating the exchange of histones and protamines through its interaction with them.

### 2.6. BAF-L KO Results in Male Subfertility

**Figure 7:**
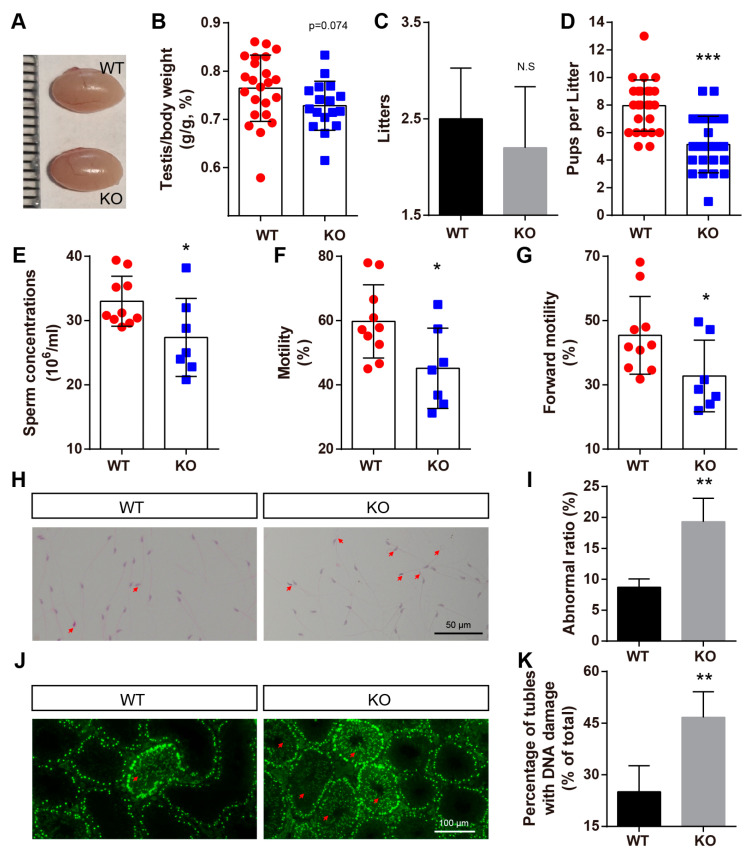
BAF-L KO results in male subfertility. (**A**) Photograph shows comparable testicular size in 8-week-old BAF-L KO mice. (**B**) Quantification shows decreased, but not significantly, ratio of testis and bodyweight of 8-week-old BAF-L KO mice. Error bars indicate SD. *n* = 24 for WT and *n* = 17 for KO. (**C**) Quantification displays comparable litters produced by BAF-L KO males and WT controls for a period of 3 months when they mate with WT females. *n* = 10. (**D**) Quantification shows significantly fewer pups per litter of BAF-L KO males than those of WT controls. Error bars indicate SD. *** *p* < 0.001 by two-tailed Student’s *t*-test. *n* = 25 for WT and *n* = 22 for KO. (**E**–**G**) Measurements show reduced sperm concentration, motility, and forward motility in 8-week-old BAF-L KO mice. Error bars indicate SD. * *p* < 0.05 by two-tailed Student’s *t*-test. *n* = 10 for WT and *n* = 7 for KO. (**H**,**I**) H&E staining and quantification reveal reduced teratozoospermic ratio in BAF-L KO mice. Red arrows indicate abnormal sperms. Error bars indicate SD. ** *p* < 0.01 by two-tailed Student’s *t*-test. *n* = 3 for WT and *n* = 4 for KO. (**J**,**K**) Immunofluorescence staining and quantification show increased γH2AX^+^ cells in the seminiferous tubules of 8-week-old BAF-L KO testes. Red arrows indicate germ cells with DNA damage. Error bars indicate SD. ** *p* < 0.01 by two-tailed Student’s *t*-test. *n* = 4.

While defects in spermiogenesis were caused by BAF-L deficiency, the testicular size of adult BAF-L KO mice seemed comparable with that of WT controls, even though the testis index was slightly decreased (Figure 7A,B). The histone–protamine exchange defects could result in abnormal spermatozoal morphology, reduced sperm motility, and male fertility [23,26,27]. As we observed defects in spermiogenesis caused by an abnormal histone–protamine exchange, we further examined the male fertility of BAF-L KO males. We crossed 6-week-old BAF-L WT or KO males with WT females and examined their produced offspring for three months. Smaller, but not significantly, litters were produced during this period by BAF-L KO male mice (Figure 7C). However, we found the number of pups per litter of KO males was notably fewer than that of WT controls, indicating the reduced fertility of BAF-L KO males (Figure 7D). This result is consistent with our findings in infertile human subjects. To better elucidate the basis for the impaired male fertility of BAF-L KO mice, sperm parameters analysis was performed. Analysis of spermatozoa collected from cauda epididymis revealed a significant reduction in sperm concentration, motility, and forward motility (Figure 7E–G), indicating a condition similar to human oligo-asthenozoospermia [28]. Besides, an increased proportion of abnormal-appearing spermatozoa was produced by BAF-L KO mice (Figure 7H,I). Impaired sperm parameters and defects in the spermatozoal number and morphology may result from spermatogenic damages, as we observed widespread DNA damage in BAF-L KO testes, as indicated by γ-H2AX immunofluorescence labeling (Figure 7J,K). All these data reveal that BAF-L is important for spermatozoal function and suggest a germ-cell-intrinsic role of BAF-L in maintaining male fertility.

## 3. Discussion

The progressive removal and replacement of histones from spermatids’ DNA by transition proteins and protamines are critical for proper spermiogenesis [25,29,30]. In this study, we report that BAF-L is a histone- and protamine-interacting protein that is involved in histone replacement to promote the spermiogenic progress. BAF-L is highly expressed in testes and is mostly concentrated in the nucleus of male germ cells and correlates with the progress of spermiogenesis. BAF-L deficiency in mice causes defects in the histone removal of elongating spermatids and disturbs the spermiogenic progress, resulting in reduced spermatozoal activity and male fertility. Our results differ from a recent study carried out by Niu et al. generating BAF-L KO mice, which only targeted exon 3 of BAF-L and showed adult BAF-L knockout mice displaying normal testicular histological structure and male fertility [31]. The different phenotypes may have resulted from the different strategy they used to generate the mice as compared to ours. We used CRISPR-Cas9 system targeting on exons 2 and 3 of the transcript variant 2 of BAF-L (NCBI Reference Sequence: NM_ 001044750.1) to delete a 9350 bp sequence, while they targeted exon 3 of the transcript variant 1 of BAF-L (NCBI Reference Sequence: NM_ 207275) to delete 253 bp. Moreover, the absence of the BAF-L protein was not validated in their study, and they mainly focused on the basic histological structure of adult testis and not the spermatogenic progress. Additionally, they examined male fertility through a mating period of a month, while we evaluated this within a much longer period.

Two homolog genes of the barrier-to-autointegration factor (BAF and BAF-L) were identified in the mammalian genome [12,13,32], but their biological functions are not well defined, especially for BAF-L. The expression pattern of these two genes is quite different. The widespread expression of BAF is detected among a significant number of mouse tissues, such as the intestine, spleen, heart, ovary, testis, etc. [33]. In contrast, Tifft et al. reported that the expression of BAF-L was highest in the pancreas and testis and lower in some other tissues such as the lung, liver, etc. [13], while Niu et al. showed the restricted expression of BAF-L only in testes [31], and all these data were obtained solely via PCR. In our study, by using qRT-PCR and Western blots with a specific antibody, we found BAF-L was most abundant in testes, and less BAF-L was detected in the pancreas and spleen. Most importantly, we demonstrated that BAF-L is a male germ cell marker that is expressed along with spermatogenic maturation, and extremely increased expression of BAF-L is observed along with spermiogenesis, which is consistent with a microarray study conducted by Maratou et al. [15]. These data suggest potential roles of BAF-L in regulating spermiogenesis.

Unlike somatic cells, over 90% of the histones that are wounded by DNA to form nucleosomes are replaced by protamines mammalian in male germ cells during spermiogenesis [7]. Complex and germ-cell-intrinsic factors and processes are involved in the exchange of histones and protamines, such as different variants of histones and its post-translational modifications, as well as their related chromatin remodelers, etc. [34]. Acetylation is one of the most important post-translational modifications of histones that is critical for regulating nucleosome assembly, gene activity, and transcription [35]. Moreover, the global hyperacetylation of histones is reported to play an essential role in the replacement of histones by transition proteins and protamines to compact the genome during spermiogenesis [7], while defects in histone acetylation result in aberrant spermatid development [34,36]. Here, in our study, we found the interaction of BAF-L with histones H3/H4 may be important for their acetylation, as we detected robustly decreased levels of acetylated histones H3/H4, which could be responsible for defects in the histone–protamine exchange in BAF-L KO testes. In addition to this, given that we also noticed an interaction of BAF-L with protamine and deficiency of BAF-L, which caused an accumulation of DNA damage in elongating/elongated spermatids, we speculate BAF-L may be implicated in the maintenance of highly condensed chromatin structures of maturing/mature spermatids to protect them from damages, the notion of which was found in its homolog gene product—BAF, which is important for the repairment of nuclear ruptures [9]. While these results have contributed to understating the biological function of BAF-L, more work need to be carried out to further address the specific mechanisms underlaying these findings. 

Our work also has implications for clinical research on male infertility. BAF-L could be a diagnostic risk factors for male infertility. In support of this theory, we found reduced expression of spermatozoal BAF-L in asthenozoospermic patients, and the expression of BAF-L was positively correlated with spermatozoal motility and forward motility. It should be noted that the expression of spermatozoal BAF-L in asthenozoospermic subjects varied significantly, which could be a consequence of the complexity of the cause of human asthenozoospermia, in which both sperm-cell-intrinsic and environment-related factors are implicated [37,38]. In addition, we have also validated this with a BAF-L knockout mouse model that had fewer sperm, lower sperm motility/forward motility, and reduced male fertility. Therefore, we conclude that male-germ-cell-specific expression of BAF-L plays an important role in maintaining male fertility.

## 4. Materials and Methods

### 4.1. Patients, Semen Collection, and Analysis

Following approvals from the Institutional Review Board of Sichuan Agricultural University and People’s Hospital of Ya’an, semen samples from patients attending sterility evaluation from September 2018 to June 2019 were collected in People’s Hospital of Ya’an. The information about the lifestyle, past illness, usage of alcohol or drugs, and pregnancy history were collected for exclusion of the subjects. Subjects with testicular cryptorchidism, varicocele, prostatitis, prostatitis, genitourinary anomalies, illicit drug use, and those who had been exposed to any environmental or occupational toxicants, those who used medication with proven toxicity to fertility, and those who had been exposed to radiation or heat were excluded from this study. After 48 h–72 h of sexual abstinence, semen samples were collected in sterile containers by masturbation, and then they were liquefied at 37 °C for 20 to 60 min. Then, sperm parameters were analyzed with the Sperm Quality Analyzer (SQA-V, Medical Electronic System, Ltd., Caesarea, Israel), and semen samples of normospermia and asthenozoospermia were collected according to WHO criteria [28]. Subjects with nonliquefaction and leukocytospermia were excluded. Finally, spermatozoa were collected from the semen samples for qRT-PCR with the methods as previously described [39]. 

### 4.2. Animals

All mouse work was carried out in accordance with the Animal Care and Use Committee guidelines of Sichuan Agricultural University. Mouse strains used in this study have a C57BL/6N background. Conventional BAF-L knockout mice (BAF-L −/−) were generated with CRISPR-Cas9 system targeting exon 2 and 3 of BAF-L (Figure 3A) (NCBI Reference Sequence: NM_ 001044750.1) by Cyagen Biosciences (Suzhou, China). gRNA target sequence are as follows: gRNA1 (matching forward strand of gene): GAGCTCACCTTCTGCTGCCGTGG; gRNA2 (matching reverse strand of gene): ATCCTCCATCCTAGTCCTAGAGG. Founder mice and their offspring were identified by genotyping PCR (WT Forward: 5′-TCC AGT ACC AGG AAT CTG CTT ATC-3′, Reverse: 5′-TGC TGT TTC AAG GAG GGA TTC-3′; CUT Forward: 5′-AAT CTC TCA CTC AGG TTC TCT TC-3′, Reverse: 5′-TGC TGT TTC AAG GAG GGA TTC-3′) [40]. In addition, the absence of BAF-L protein was validated with affinity-purified rabbit polyclonal BAF-L antibody that was generated by immunizing rabbits with full-length GST fusion protein of mouse BAF-L. All mice were bred under SPF conditions in standard individually ventilated cages at 20–22 °C, with a 12 h light–12 h dark cycle, 50–70% humidity, and ad libitum access to standard chow and water. 

### 4.3. In Situ Hybridization

Fresh testes were fixed with a fixative buffer for in situ assay (G1113-500, Servicebio, Wuhan, China), and paraffin sections were then prepared 12 h after the fixation and mounted on sides to heat to 62 °C for 2 h. Two treatments with xylene (15 min) and two with 100% ethanol (15 min) were used to remove the paraffin. The sections were air-dried and immersed in DEPC water, followed by boiling for 10–15 min in retrieval solution. Then, the proteinase K (G1205, Servicebio, Wuhan, China) was used to digest the cooled sections for 20 min at 37 °C, and the sections were washed with DEPC water first, followed by 3 times with PBS. The incubation of the slides was performed with a prehybridization solution (G3016-3, Servicebio, Wuhan, China) for 1 h at 37 °C, and then the digoxigenin-labeled probes (5′-DIG-CAC ATC CTT TTC CCC AAT GGG TTC AGA GA-DIG-3′), which were designed according to the exon 3 (deleted in KO) of mouse BAF-L gene, were added to the sections and incubated overnight at 37 °C after gently removing the prehybridization solution. After this, the slides were washed with 2 × SSC for 10 min at 37 °C, followed by 1 × SSC for 5 min at 37 °C (twice) and 0.5 × SSC for 10 min at RT. The 3% BSA solution (G5001, Servicebio, Wuhan, China) was used to block the slides for 30 min at RT. After removing the blocking solution, the anti-DIG-AP solution (200-052-156, Jackson) was used to incubate the slides for 40 min at 37 °C. The slides were washed with TBS for 5 min (four times), and then the color development was performed with BCIP/NBT buffer (AR0043, Boster, Wuhan, China). Finally, the slides were mounted in neutral balsam (G8590, Solarbio, Beijing, China) and photographed with a microscope (BX61VS, Olympus, Tokyo, Japan).

### 4.4. Quantitative Realtime PCR

TRizol reagent (15-596-026, Invitrogen, Waltham, MA, USA) was used to extract the Total RNA from testes using according to previous description [41]. Then, reverse transcription was performed with ~1 μg total RNA according to manufacturer’s instructions (RR047A, Takara, Kusatsu, Shiga, Japan). Quantitative real-time PCR was performed with a Bio-Rad CFX96 system (Bio-Rad, Hercules, CA, USA), and the relative gene expression was normalized to internal control as β-Actin. Primers used in this study are listed below: mouse β-actin Forward: 5′-AGAGGGAAATCGTGCGTGAC-3′, Reverse: 5′-CAATAGTGATGACCTGGCCGT-3′; mouse BAF-L Forward: 5′-GTTTCAACAAGGCCTATGTCCTGC-3′, Reverse: 5′-CTATAGGAAACAGGAGCACC-3′; mouse Tnp1 Forward: 5′-GAGAGGTGGAAGCAAGAGAAAA-3′, Reverse: 5′-CCCACTCTGATAGGATCTTT GG-3′; mouse Tnp2 Forward: 5′-GAAGGGAAAGTGAGCAAGAGAA-3′, Reverse:5′-GCA TAG AAATTGCTGCAGTGAC-3′; mouse Prm1 Forward: 5′-ACA AAATTCCAC CTGCTCACA-3′, Reverse: 5′-GTTTTTCATCGGCGGTGGC-3′; mouse Prm2 Forward: 5′-GCTGCTCTCGTAAGAGGCTACA-3′, Reverse: 5′-AGTGATGGTGCCTCCTACAT TT-3′.

### 4.5. Western Blotting

Total protein was extracted from tissues by mincing them with scissors in tissue lysis buffer (2% SDS with proteinase inhibitors and phosphatase inhibitor). The protein concentration was measured with BCA Protein Assay Kit (23225, Thermo Scientific, Waltham, MA, USA). For Western blotting, 5–10 µg of protein was loaded into SDS-PAGE gels and blotted with antibodies listed in Table 1, according to standard Western blotting procedures as reported before [42].

### 4.6. Immunochemical Staining

Immunohistochemical staining was performed according to the manufacturer’s instructions for SABC-POD Kit (Boster, SA2002, Wuhan, China). Briefly, paraffin-embedded sections were first deparaffinized and rehydrated, and 3% H_2_O_2_ was used to block the endogenous peroxidase activity for 20 min at RT. The slides were washed with PBS, before the antigen retrieval was performed with Citrate Buffer (pH = 6.0) under high pressure. Blocking buffer (10% donkey serum in PBS + 0.1% Triton X-100, if a permeabilization was needed) was used to block the tissues sections for 1 h at RT. Primary antibodies, diluted in PBS with 1% donkey serum, were incubated on the slides at 4 °C overnight. After three washes with PBS, slides were then incubated with the biotin-labeled secondary antibody and SABC in turn at RT for 30 min. Finally, the slides were developed with color by DAB and counterstained with hematoxylin (if needed).

### 4.7. Immunofluorescence Staining

Paraffin-embedded slides were first deparaffinized and rehydrated, and the antigen retrieval was performed with Citrate Buffer (pH = 6.0) under high pressure. Tissue sections were then blocked with blocking buffer (1X PBS + 10% donkey serum + 0.01 g/mL BSA + 0.1% Triton X-100) at room temperature for 60 min. Diluted primary antibodies (in PBS with 1% donkey serum) were added to the slides and incubated at 4 °C overnight. The slides were washed with PBS three times for 15 min, and then they were incubated with secondary antibodies in darkness at RT for 90 min. Another three washes in PBS were performed before the slides were mounted by coverslips with Prolong Gold with DAPI mounting medium (P36962, Invitrogen, CA, USA) and photographed with a microscope (BX61VS, Olympus, Tokyo, Japan).

### 4.8. H&E Staining

Modified Davidson’s Fixative solution was used to fix the fresh testes (4 °C for 24 h), which were then stored in 70% ethanol. Samples were embedded in paraffin and sectioned (5 μm), and the sections were mounted on slides (poly-L-lysine coated) followed by dewaxing and rehydration. Finally, hematoxylin and eosin (H&E) staining was performed according to the manufacturer’s instructions (G1120 for H&E, Solarbio, Beijing, China).

### 4.9. Sperm Parameters Analysis 

Caudal epididymal sperms of adult mice were collected by placing minced cauda epididymis from both sides into Biggers–Whitten–Whittingham solution (BWW) (1 mL, 37 °C prewarmed) (G2586, Solarbio, Beijing, China), in which sperms could swim out and stay alive for hours [43]. The sperm count, concentration, motility, and forward motility were analyzed using a Fully Automatic Sperm Quality Analyzer (BX-9100A, Baoxing Medical Equipment, Xuzhou, China), after an incubation of the solution for 30 min at 37 °C. To analyze the sperm morphology, 10 ul of sperm-containing BWW solution was smeared on poly-l-lysine-coated slides and aired dry. Then, H&E staining was performed with methanol-fixed sperm smear, and the abnormal sperms were finally quantified with the photographed images taking by a microscope (BX61VS, Olympus, Tokyo, Japan).

### 4.10. Cell Culture

TM3 and TM4 cells were obtained from the National Infrastructure of Cell Line Resource (Beijing, China). The cells were validated with short tandem repeat (STR) analysis before use and maintained with DMEM (11995, Solarbio, Beijing, China) containing 10% fetal bovine serum in an incubator under an atmosphere of 5% CO_2_ at 37 °C.

### 4.11. Immunoprecipitation

Immunoprecipitation was performed according to a previous study [44]. Briefly, ~25 mg of testicular tissue was ultrasonically minced with 250 µL of lysate buffer (50 mM Tris, 150 mM NaCl, 0.1–0.5% detergent (Tween 20, T8220, Solarbio, Beijing, China), pH 7.5 containing protease inhibitor cocktail, and 1 mM PMSF) on ice. Supernatant was collected after a centrifugation performed at 14,000× *g* for 10 min, at 4 °C. A total of 50 µL of the supernatant was selected as the input sample. Protein A/G magnetic beads (Bimake, B23202, Shanghai, China) were used to immunoprecipitated with 5 µg/mL antibody and 200 µL of testicular tissue lysates for 1 h at 4 °C, respectively. The immune complex was washed three times with washing buffer (50 mM Tris, 150 mM NaCl, 0.1–0.5% detergent (Tween 20), pH 7.5) and resuspend in 1 × SDS-PAGE loading buffer. Finally, individual proteins were detected as described for Western blotting.

### 4.12. Statistical Analysis

Data represent the mean ± standard deviation (SD) or mean ± standard error of the mean (SEM). Two-tailed Student’s *t*-test was performed for all statistical significance analysis using GraphPad Prism software (Version 6.0, San Diego, CA, USA ). * *p* < 0.05, ** *p* < 0.01, *** *p* < 0.01.

## 5. Conclusions

In summary, this study validates a prominent role of BAF-L in spermiogenesis and maintaining spermatids’ histones–protamines homeostasis, which is important for male fertility. Reduced spermatozoal BAF-L expression links to human male infertility, suggesting BAF-L as a diagnostic and therapeutic target for certain male infertility conditions.

## Figures and Tables

**Table 1:** Antibodies used in this study.

Antibodies	Source	Identifier/Application
GATA-4(D3A3M) Rabbit mAb	Cell Signaling Technology	Cat#36966, IHC: 1:800
SCP3 Rabbit pAb	Abcam	ab15093, IHC: 1:500
ACRV1 Rabbit pAb	Proteintech	Cat#14040-1-AP, IHC: 1:500
pCAM Rabbit mAb	ABclonal	Cat#A19301 IHC: 1:100, WB: 1:500
PRM1 Rabbit pAb	Proteintech	Cat#15697-1-AP WB: 1:1000
BAF-L Rabbit pAb	Generated by immunizing rabbits with full-length GST fusion protein of mouse BAF-L	IHC: 1:200, IP: 1:100, WB: 1:1000
Histone H4 Rabbit mAb	ABclonal	A19815, IF: 1:100, IP: 1:100, WB: 1:1000
Histone H3 Rabbit mAb	ABclonal	A17562, IF: 1:100, IP: 1:100, WB: 1: 1000
Histone H2A Rabbit mAb	ABclonal	A3692, IF: 1:100, WB: 1:1000
Histone H2B Rabbit mAb	ABclonal	A19812, WB: 1:1000
Ac-Histone H4 (E-5) Mouse mAb	Santa Cruz	sc-377520, WB: 1:500
Ac-Histone H3 (AH3-120) Mouse mAb	Santa Cruz	sc-56616, WB: 1:500
Phospho-Histone H2A.X(Ser139) (20E3) Rabbit mAb	Cell Signaling Technology	Cat#9718, IF: 1:400

## Data Availability

Source data are provided in this paper and are available from the corresponding author upon reasonable request.
